# Evaluation of FcγRIIIB-NA1/NA2 Polymorphism in Visceral Leishmaniasis

**DOI:** 10.5812/ircmj.12437

**Published:** 2014-04-05

**Authors:** Mohammad Abasi, Pegah Lotfi, Ahad Bazmani, Mohamad Matini, Mehrdad Hajilooi

**Affiliations:** 1 Faculty of Medicine, Shahid Beheshti Hospital, Hamadan University of Medical Sciences, Hamadan, IR Iran; 2 Department of Microbiology, Faculty of Medicine, Hamadan University of Medical Sciences, Hamadan, IR Iran; 3 Infectious and Tropical Diseases Research Center, Tabriz University of Medical Sciences, Tabriz, IR Iran; 4 Department of Parasitology, Faculty of Medicine, Hamadan University of Medical Sciences, Hamadan, IR Iran; 5 Department of Immunology, Faculty of Medicine, Hamadan University of Medical Sciences, Hamadan, IR Iran

**Keywords:** Polymorphism, Genetic, Leishmaniasis, Visceral, Iran

## Abstract

**Background:**

Several lines of evidence demonstrating that innate and adaptive immunity play important roles in the defense against visceral leishmaniasis (VL). A polymorphism within the FcγRIIIB gene can lead to the expression of three variants of NA1, NA2, and the combined one (NA1/NA2) which alters affinity of IgG to its receptor.

**Objectives:**

The main aim of this study was to evaluate the FcγRIIIB-NA1/NA2 polymorphism in the FcγRIIIB gene of VL patients in comparison to healthy controls.

**Patients and Methods:**

In this cross-sectional study, three groups; 54 seropositive patients with clinical presentation of VL (group 1), 104 seropositive patients without clinical presentation (group 2), and 104 healthy controls (group 3) were evaluated with respect to the FcγRIIIB-NA1/NA2 polymorphism using a PCR-SSP method. The titration of anti-leishmania antibodies was analyzed using an immunoflorescence technique.

**Results:**

Our results indicated that polymorphisms within the FcγRIIIB gene (that lead to the expression of the NA1/NA2 isoforms) are significantly associated with VL. The results demonstrated that the genotype heterozygotic for FcγRIIIB-NA1/NA2 expression was significantly increased in VL patients, group 1 when compared to groups 2 and 3. Conversely, there is a decrease in homozygous NA1 and NA2 genotypes in VL patients; however, the overall frequency of NA1 and NA2 alleles appear similar across the three cohorts examined.

**Conclusions:**

According to our results, it is likely that the increased frequency of the FcγRIIIB-NA1/NA2 genotype is associated with impaired immune responses against VL and its subsequent clearance from the patient.

## 1. Background

Various Leishmania species are responsible for leishmaniasis, which is a vector-borne parasitic disease (1). Although, the sand fly is the major vector of the leishmaniasis transfer into humans (1), the parasite can also be transmitted via needle stick injury (2), blood transfusions (3) or through placenta during pregnancy (4). Several studies identified that the immune responses against leishmaniasis is dependent on genetic status, environmental factors and the infecting species (5). Clinical manifestations of Leishmaniasis are various including; cutaneous, mucocutaneous, and visceral (6) which is the most severe and lethal form if left untreated. The visceral form (VL) is also known as Kala Azar (7).

It seems that the progression and severity of the disease is based on several host factors which include a genetic component. The final clinical outcome and severity of the disease is, in part, determined by how well the host immune system can respond to the infection. Previous studies reported that both innate or adaptive immunity play important roles in both defense against VL and the eradication of the parasite (8). Neutrophils which are important innate immunity cells play critical roles combating leishmaniasis via phagocytosis of the parasite, degranulation, and generation of reactive oxygen species (ROS).

The mechanism of internalization of the parasite functions in an antibody/Fc-gamma receptor dependent manner with FcγRIIIB contributes a major role in the process (9). Therefore, the efficacy between FcγRIIIB and its corresponding antibodies (IgG) leads to appropriate immune responses against VL. Previous studies have showed that the FcγRIIIB gene (NM-00570.4) contains a polymorphism including FcγRIIIB*1 and FcγRIIIB*2 alleles, which code for human neutrophil antigen (HNA)-1a (or NA1) and HNA-1b (or NA2) (10). Interestingly, studies demonstrated that FcγRIIIBNA1/NA1 is associated with phagocytosis of IgG1 and IgG3-coated particles more powerful than the FcγRIIIB-NA2/NA2 genotype (11).

## 2. Objectives

Based on the fact that, the quality and quantity of immune responses determine the outcome of leishmaniasis, the main aim of this study was to evaluate the FcγRIIIB-NA1/2 polymorphism in seropositive VL patients with and without clinical presentation in comparison with healthy controls.

## 3. Patients and Methods

### 3.1. Subjects

This cross-sectional study was performed on 3 groups; 54 patients with clinical presentation of VL who were seropositive for the *Leishmania* (group 1), 104 seropositive patients without clinical presentation (group 2) and finally, 144 healthy controls whose age, sex, nationality and race were matched (group 3). The patients were selected from East Azarbaijan (a city located in northwest of Iran) where the *Leishmania infantum* is endemic (12-14), while, other species were not reported. Visceral leishmaniasis was diagnosed by an expert specialist according to medical history, laboratory findings, and clinical presentations. The study protocol was approved by local Ethical Committee of the Ardabil University of Medical Sciences. An informed consent form was filled out and signed by participants prior to entrance in the study.

### 3.2. DNA Extraction

Peripheral blood was obtained from the participants in EDTA pre-treated tubes and genomic DNA was purified using a commercial kit (Bioneer, Korea) according to the manufacturer’s guidelines. The purified DNA was aliquoted and stored at -20˚C for use in PCR-SSP analysis.

### 3.3. Polymorphism Detection

The FcγRIIIB gene polymorphism (within exon 3) was evaluated using PCR-SSP method. PCR was performed in a final volume of 20 μL containing 2 μL of Taq DNA polymerase buffer (10X), 0.3 μL of Taq DNA polymerase (five units), 1 μL of each dNTP [(dATP, dCTP, dGTP, dTTP) at a concentration of 10 mM, 2 μL of each primer (25 ng/μL), 2 μL of MgCl_2_ (stock concentration 1.5 mM), 1 μL of prepared DNA and sterile double distilled DNase free water. 

The sequences of the forward and reverse primers for amplification of the NA1 allele were 5'-CAGTGGTTTCACAATGTGAA-3' and 5'-CATGGACTTCTAGCTGCACCG-3' and for the NA2 allele were 5'-CTCAATGGTACAGCGTGCTT-3' and 5'-CTGTACTCTCCACTGTCGTT-3'. The PCR product sizes of NA1 and NA2 genes were 142 and 169 bp, respectively. The amplification was performed using the following procedure: 95˚C for 5 minutes (denaturation) followed by 35 cycles of 30 seconds at 95˚C, 53˚C for 30 seconds and 72˚C for 40 seconds using a thermal cycler (Bioneer, South Korea). Electrophoresis was performed on a 2.5% pretreated ethidium bromide agarose gel (Cinnaclon-Iran) for the detection of PCR products.

### 3.4. Immunoflorescence Assay

The titration of the anti-leishmania antibody was performed using a commercial kit (Qiagen, USA) according to the manufacturer’s guidelines.

### 3.5. Statistical Analysis

Hardy-Weinberg equilibrium analysis was used to check the validity of the raw data. The χ^2^ and One-way analysis of variance (ANOVA) tests were used from the SPSS software version 13 to determine the differences between groups and a P value less than 0.05 was considered as significant.

## 4. Results

Our results indicated that the FcγRIIIB-NA1/NA2 polymorphisms were significantly associated with VL (Table 1). The results demonstrated that the FcγRIIIB-NA1/NA2 genotype was significantly higher in group 1 compared to groups 2 and 3 (Table 1). The statistical analysis revealed that the difference between groups regarding the genotypes were significant (P = 0.02). However, the results showed that the groups were not different regarding the frequency of the FcγRIIIB-NA1 and NA2 alleles (Table 1) (P = 0.17). The current results revealed that age was not associated with antibody production against leishmania (P = 0.07). Our results also demonstrated that women had higher antibody titers against leishmania than men (P =.006) (Table 2). The current results also demonstrated that the VL patients (group 1) carrying the FcγRIIIB-NA1/NA1, NA2/NA2 and NA1/NA2 genotypes had mean anti-leishmania antibody titrations values to the 2.75 ± 0.41, 2.47 ± 1.63, and 2.34 ± 0.43 respectively, which were not statistically significant (P = 0.82). Our results revealed that participants in group 2 carrying the FcγRIIIB-NA1/NA1, NA2/NA2 and NA1/NA2 genotypes had mean anti-leishmania antibody titrations values to the 3.5 ± 0.17, 2.9 ± 0.09, and 3.2 ± 0.06, respectively. The statistical analysis demonstrated that the difference between group 2 carrying the FcγRIIIB-NA1/NA1 and NA2/NA2 was significant (P = 0.003).

**Table 1.** The Prevalence of FcgRIIIB Polymorphisms Among Patients ^a^

	Group 1 ^b^	Group 2^b^	Group 3 ^b^	P value ^c^
**Genotypes**				0.024
NA1/NA1	8 (14.8)	21 (20.2)	13 (9)	
NA2/NA2	23 (42.6)	58 (55.8)	79 (54.9)	
NA1/NA2	23 (42.6)	25 (24)	52 (36.1)	
**Alleles**				0.176
NA1	39 (36.1)	67 (32.2)	78 (27.1)	
NA2	69 (63.9)	141 (67.8)	210 (72.9)	

^a^ Data in table are presented as No. (%).

^b^ Group 1: Patients with clinical presentation of VL and seropositive for the leishmania, Group 2: Without clinical presentation but seropositive, Group 3: Healthy controls.

^c^ Significance (P values) is shown for the comparison between groups 1 and 2.

**Table 2.** The Anti-Leishmania Antibody Titration in Women and Men ^a^,^b^

Titration of Anti-Leishmania Antibody	Sex		Total
	Female	Male	
**0**	89 (47.6)	62 (53.9)	151 (50)
**1/10**	2 (1.1)	9 (7.8)	11 (3.6)
**1/20**	6 (3.2)	5 (4.3)	11 (3.6)
**1/40**	65 (34.8)	27 (23.5)	92 (30.5)
**1/80**	19 (10.2)	6 (5.2)	25 (8.3)
**1/160**	4 (2.1)	6 (5.2)	10 (3.3)
**1/320**	2 (1.1)	0	2 (0.7)
**Total**	187 (100.0)	115 (100.0)	302 (100.0)

^a^ Data are presented in No. (%).

^b^ The table illustrates that the percent of women who have a high titer (1/40, 1/80 and 1/320) of anti-Leishmania antibodies were more than men.

## 5. Discussion

Previous studies demonstrated that FcγRIIIB binds to IgG subclasses and its affinity to IgG is regulated by the FcγRIIIB isoforms (i.e. NA1 or NA2) which are in turn determined by polymorphisms within the gene (9). Our results showed that FcγRIIIB-NA1/NA2 genotype was significantly higher in group 1 (VL and seropositive) compared to group 2 and 3, while, FcγRIIIB-NA1/NA1 and NA2/NA2 genotypes in group 2 were higher than group 1. Therefore, it appears that FcγRIIIB-NA1/NA2 genotype is associated with VL and may be considered as a risk factor for developing VL in Iranian patients infected with *Leishmania*. Based on the fact that the FcγRIIIB-NA1 isoform for human IgG2 and IgG3 has a higher affinity than the FcγRIIIB-NA2 isoform (9) and our results which revealed that the FcγRIIIB-NA2/NA2 genotype was more frequent in group 2, it may be concluded that the rate of FcγRIIIB expression but not its affinity to IgG2 and IgG3 can determine the outcome of leishmaniasis. Thus, it seems that the prevalence of FcγRIIIB-NA1/NA2 genotype in VL patients may be a risk factor for *Leishmania*.

Our results may propose a mechanism for inadequate immune responses against *Leishmania* in VL patients. Accordingly, previous investigations demonstrated that FcγRIIIB is expressed on the natural killer (NK) cells and participates in antibody dependent cell-mediated cytotoxicity (ADCC) against infected cells (15, 16). Therefore, it appears that the polymorphisms within FcγRIIIB gene can affect functions of NK cells which is reported by previous studies (17, 18). To the best of our knowledge this is the first study which evaluated the FcγRIIIB-NA1/NA2 polymorphisms in patients with VL.

But several studies are conducted on the malaria which is another parasitic infection (19). For example, Adu et al. revealed that FcγRIIIB-NA2 allele was significantly associated with clinical malaria in Ghanaian children (20). Ouma et al. reported that FcγRIIIB-NA1 allele was associated with reduced risk of severe malarial anemia (SMA), while, the patients carrying FcγRIIIB-NA2 allele had increased susceptibility to SMA (21). Also, Omi et al. also identified that FcγRIIIB-NA1/NA2 polymorphism have interactive effects on immune responses against malaria infection (22). Therefore, based on our data and the aforementioned studies it may be concluded that the FcγRIIIB-NA1/NA2 genotype is significantly associated with parasite infections including leishmaniasis and malaria.

In contrast with our results the polymorphisms in other FcγRs including FcgRIIA were not associated with VL. For example, our previous study revealed that polymorphism in the FcgRIIA gene was not associated with Iranian VL patients (unpublished data). Similarly, Oliveira et al. also reported that the polymorphism in the FcγRIIIB gene was not associated with American tegumentary leishmaniasis (ATL) (9). Their results also demonstrated that the polymorphism was not related to the development of various clinical forms of ALT (9).

According to our results and those of Oliveira et al. (9) it may be concluded that the FcγRIIIB (but not other FcγRs) polymorphisms play important roles in VL pathogenesis. Our results also identified that age was not associated with antibody production against *Leishmania*. Therefore, it may be concluded that age does not influence humoral immunity against leishmaniasis. Additionally, our results showed that the FcγRIIIB-NA1/NA2 polymorphism was not associated with anti-*Leishmania* antibody titration in VL patients but FcγRIIIB-NA1/NA1 genotype was associated with higher antibody production in seropositive participants without clinical presentation. Hence, based on our results and previous studies it may be concluded that FcγRIIIB-NA1/NA1 genotype can affect either IgG/receptor affinity or antibody production against the *Leishmania* parasite in Iranian population. Furthermore, the results showed that women produced higher antibody against *Leishmania* than men. Based on the different hormonal status, the immune system of women shift to the Th2 system (23), so, this population has stronger humoral immunity than men. Therefore, the higher antibody titration against *Leishmania* in women may be related to the different hormonal status.

Finally, based on our results it appears that the FcγRIIIB-NA1/NA2 genotype is significantly associated with Iranian VL patients and more studies which evaluate other FcγRs are recommended for future studies.
